# Spontaneous Coronary Artery Dissection Following SARS-CoV-2-Associated Multisystem Inflammatory Syndrome

**DOI:** 10.7759/cureus.26479

**Published:** 2022-07-01

**Authors:** Anthony M Pettinato, Feria A Ladha, Jan Zeman, Joseph J Ingrassia

**Affiliations:** 1 Medicine, University of Connecticut School of Medicine, Farmington, USA; 2 Department of Internal Medicine, University of Connecticut School of Medicine, Farmington, USA; 3 Interventional Cardiology, Hartford Hospital, Hartford, USA

**Keywords:** myocardial infarction, spontaneous coronary artery dissection, mis-a, multisystem inflammatory syndrome, covid-19

## Abstract

Spontaneous coronary artery dissection (SCAD) is an underdiagnosed cause of acute coronary syndrome, myocardial infarction, and sudden cardiac death. During the coronavirus disease 2019 (COVID-19) pandemic, a multisystem inflammatory syndrome (MIS) emerged that is incompletely understood. While the involvement of numerous organ systems has been described, the potential cardiovascular manifestations, such as myocarditis, arterial thrombosis, or SCAD, are particularly worrisome. Here, we present a case of MIS that was preceded by an unremarkable case of COVID-19 and followed by the development of SCAD. This case highlights the importance of furthering our understanding of the potential sequelae of COVID-19 and of the potential relationship between SCAD and MIS.

## Introduction

SARS-CoV-2, the virus responsible for the coronavirus disease 2019 (COVID-19) pandemic, is now widely known for its global role in pneumonia and acute respiratory distress [1]. The delayed and long-term complications of contracting SARS-CoV-2 have been described [2]. Foremost among these is a multisystem inflammatory syndrome (MIS), which is defined by the presence of fever, multi-organ dysfunction, and elevated inflammatory markers in the context of recent SARS-CoV-2 infection and the absence of other plausible diagnoses [2]. Pertinent to both children (MIS-C) and adults (MIS-A), MIS can produce heterogeneous cardiovascular, dermatologic, gastrointestinal, and neurologic manifestations [3,4], putting patients at risk of life-threatening sequelae. Of particular concern are the cardiovascular complications, which carry high mortality and are not well-described in adults [5]. Here, we describe a case of spontaneous coronary artery dissection (SCAD), complicated by an apical aneurysm, following presumed MIS-A.

## Case presentation

A 43-year-old female with a past medical history of PCR-positive SARS-CoV-2 infection one month prior presented to the emergency department with a five-day history of fever, generalized weakness, dysphagia, vomiting, diarrhea, and a maculopapular rash on her trunk and extremities. She was found to have acute renal failure, lactic acidosis, and hypotension with leukocytosis and elevated inflammatory markers (ferritin, CRP, and D-dimer). Workup was negative for an acute infectious etiology, including new SARS-CoV-2 infection, but further revealed evidence of left-sided colitis, oral candidiasis, and new antibody-negative hypothyroidism. Given her recent SARS-CoV-2 infection in the context of rash, shock, elevated inflammatory markers, and lack of likely alternative etiology [3], she was diagnosed with presumed MIS-A and rapidly improved following treatment with corticosteroids. She was not vaccinated against SARS-CoV-2 at the time of presentation. She was discharged on a taper of oral corticosteroids and started on levothyroxine for her new hypothyroidism.

Two months later (Table 1), she presented to the emergency department with a one-week history of intermittent substernal chest tightness associated with palpitations at rest lasting several minutes before spontaneous resolution. On the day of presentation, these symptoms become more severe. In the emergency department, she only reported mild residual chest pain and nausea. Upon physical exam, she was afebrile, tachycardic, hypotensive, and had warm and well-perfused extremities. Serial ECGs demonstrated dynamic changes, with the initial ECG demonstrating left axis deviation and anterior precordial T wave inversions, while a repeat ECG two hours later demonstrated normalization of T waves in V1-V4 and ST depressions in V5-V6. CT angiography of the chest did not identify evidence of pulmonary embolism. Labs demonstrated elevated troponin, D-dimer, and LDL, and the patient was treated with nitroglycerin, aspirin, heparin, morphine, ondansetron, and intravenous fluids before transfer to a tertiary care center for further cardiac workup of likely non-ST elevation myocardial infarction (NSTEMI). The patient tested negative for SARS-CoV-2 infection at this time, and inflammatory markers were unremarkable.

**Table 1:** Summary of events.

Timeline	Event summary
History	Unremarkable SARS-CoV-2 infection 3 months prior
	MIS-A managed with corticosteroids 2 months prior
Pre-admission/ED	Intermittent substernal chest tightness with palpitations for one week
	Initial ECG with left axis deviation and T wave inversions in V1-V4
	Repeat ECG with normalization of T waves in V1-V4, ST depressions in V5-V6, and T wave blunting in aVL
	Elevated troponin and D-dimer
	CT angiography of the chest with no evidence of pulmonary embolism
	Treated with nitroglycerin, aspirin, heparin, morphine, ondansetron, and intravenous fluids
Admission (day 1)	Transferred to tertiary care hospital for management of myocardial infarction
	Continued chest pain and two episodes of vomiting
	Treated with nitroglycerin, aspirin, heparin, clopidogrel, atorvastatin, and carvedilol
	Repeat ECG with premature atrial complexes and ST elevation in V2-V4 with T wave inversions
	Repeat troponin uptrending
	Bedside echocardiography with estimated EF of 45-49% with wall motion abnormalities
	Cardiac catheterization revealed type I SCAD of the LAD and diagonal systems with TIMI I/II flow, as well as evidence of intramural hematoma and dissection flap
	Heparin and clopidogrel were discontinued, continued on aspirin, atorvastatin, and carvedilol
Day 2	Comprehensive echocardiography with quantitative EF of 39%, elevated left atrial filling pressure, an apical aneurysm, possible apical thrombus, and sustained wall motion abnormalities
	Medical regimen optimized to include aspirin, clopidogrel, carvedilol, metoprolol, spironolactone, lisinopril, atorvastatin, and warfarin
Day 3	Episodes of non-sustained ventricular tachycardia that responded to high-dose carvedilol
	Otherwise asymptomatic
Day 4	Discharged on aspirin, clopidogrel, carvedilol, metoprolol, spironolactone, lisinopril, atorvastatin, and warfarin
	Recommended outpatient cardiology follow-up in one week

**Figure 1:**
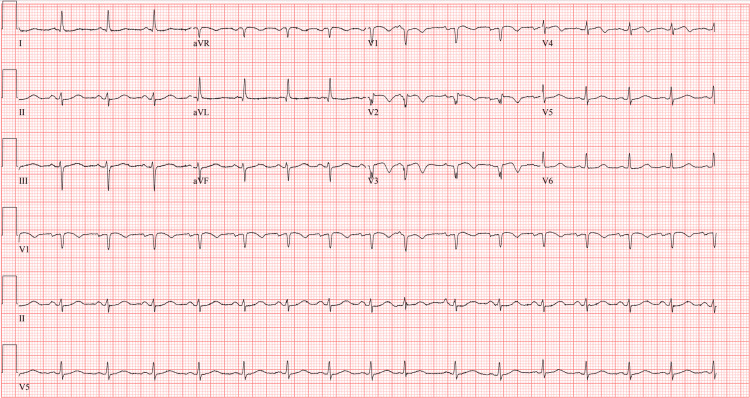
ECG performed at tertiary care center demonstrating sinus rhythm with a rate of 96 bpm, left axis deviation, premature atrial complexes, ST elevations in V2-V4, and T wave inversions in V1-V4. Concerning for an anteroseptal infarct.

**Figure 2:**
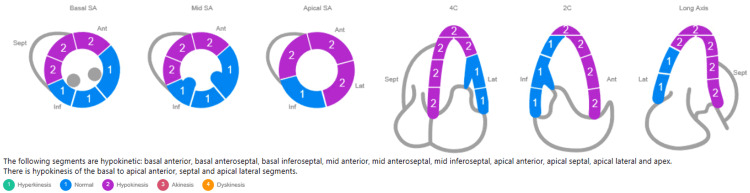
Limited bedside echocardiography demonstrated a mild decrease in left ventricular systolic function with an estimated ejection fraction of 45-49%. Hypokinesis was observed of the basal to apical anterior, septal and apical lateral segments. Wall motion score index was 1.59.

**Figure 3:**
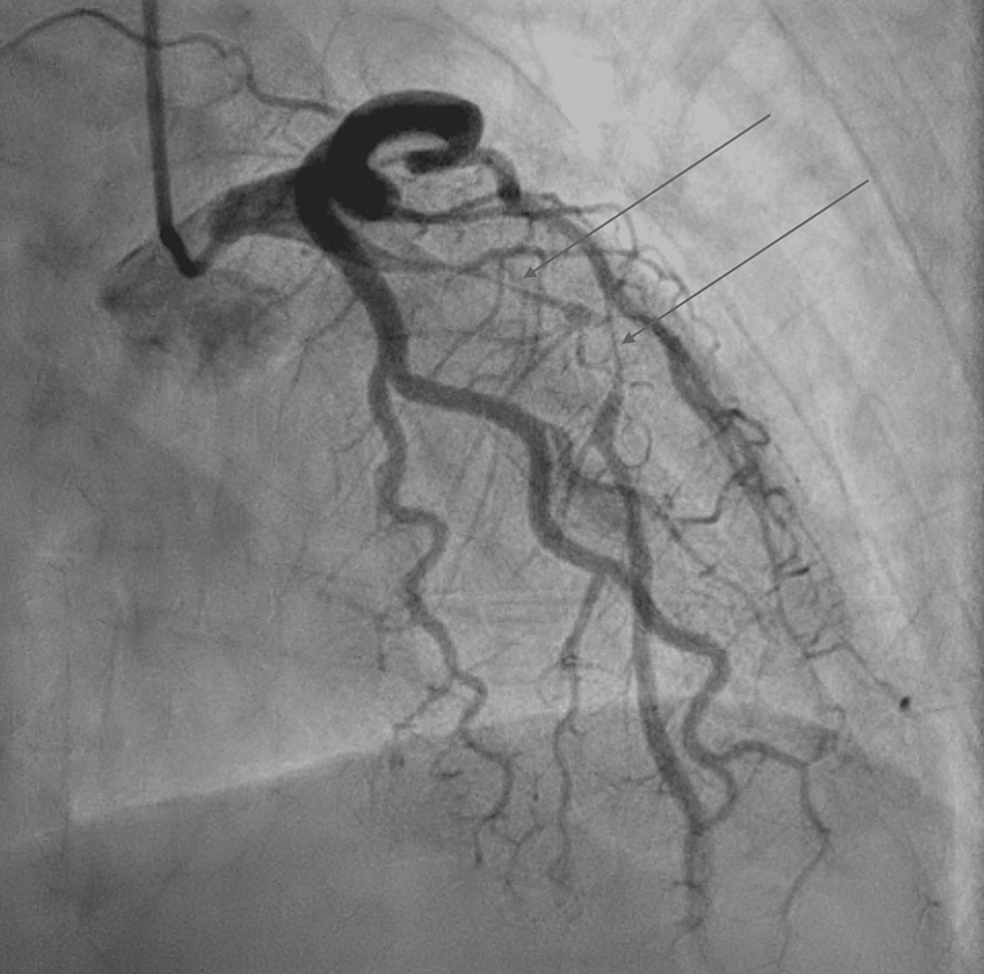
Coronary angiogram in right anterior oblique view with caudal angulation demonstrating type IIa dissection (see arrows) of the left anterior descending artery.

Upon arrival at the tertiary care center, the patient still exhibited chest pain and dynamic ECG changes, now demonstrating ST elevation in V2-V4 with T wave inversions (Figure 1), concerning an acute anteroseptal infarct. She was started on a heparin drip, aspirin, clopidogrel, atorvastatin, and beta-blocker therapy. Repeat laboratory tests revealed upward trending troponin and limited bedside echocardiography demonstrating a mildly decreased estimated ejection fraction (45-49%) with hypokinesis of the basal to apical anterior, septal and apical lateral segments of the left ventricle (LV) (Figure 2). Cardiac catheterization revealed a type IIa SCAD of the proximal to mid-left anterior descending (LAD) artery and diagonal systems with TIMI I/II flow and dissection flap in the mid LAD (Figure 3).

**Figure 4:**
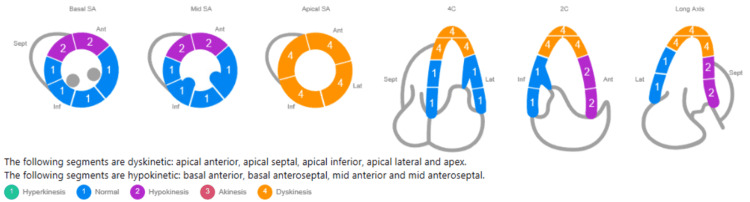
Comprehensive bedside echocardiography demonstrated moderately decreased left ventricular systolic dysfunction via quantitative EF of 39% (modified Simpson biplane). An aneurysm was present in the apex, as was a possible thrombus. Hypokinesis was observed of the anterior and anteroseptal segments, as well as dyskinesis of the apical segments. Wall motion score index was 2.12.

Given the patient’s high risk for dissection propagation with percutaneous intervention, medical management was recommended by the interventional cardiology and cardiothoracic surgery teams. Her heparin was discontinued and dual antiplatelet therapy (DAPT) was held overnight to ensure the patient remained stable. The following morning, comprehensive echocardiography with doppler demonstrated a quantitative EF (modified Simpson biplane) of 39%, an apical aneurysm, possible apical thrombus, and sustained wall motion abnormalities (Figure 4). Her medical regimen was subsequently optimized with aspirin, clopidogrel, metoprolol, spironolactone, lisinopril, and atorvastatin to manage her SCAD and reduced EF, as well as warfarin for her apical thrombus. Over the next two days, she was relatively asymptomatic apart from occasional episodes of non-sustained ventricular tachycardia, which responded to increased carvedilol dosage. After a total hospital course of four days, the patient was discharged on the above medication regimen and recommended to follow-up with outpatient cardiology.

The patient was seen in the cardiology clinic one week following discharge, at which time she reported no symptoms. At one month following admission, an echocardiogram showed recovery of her left ventricular ejection fraction to 60% and no evidence of apical aneurysm nor LV thrombus, at which time warfarin was discontinued.

## Discussion

While cases of SCAD have been reported in the context of COVID-19 infection, they have largely occurred in the immediate acute phase of the infection [6-8], whereas cases of SCAD occurring in the post-acute phase are not as widely described [2,9]. Furthermore, cases specifically associated with MIS are particularly scarce. Given the ongoing global prevalence of COVID-19, understanding and anticipating its long-term consequences is imperative to improving patient outcomes. Here, we report a case of MIS-A in a middle-aged woman with a recent COVID-19 infection who later developed SCAD complicated by an apical aneurysm and bouts of non-sustained ventricular tachycardia.

We believe that our case presents a few learning points. First, our patient’s recent history was notable for an unremarkable course of acute COVID-19, in which she was mildly symptomatic and only presented for serological testing due to a known exposure. Given that severe acute cases of COVID-19 are associated with a high risk for post-acute COVID-19 manifestations [2], our patient’s subsequent development of serious complications demonstrates how even mild cases of COVID-19 may pose a risk for serious outcomes. Our patient’s presentation one month following initial infection met the criteria for MIS-A, as defined by the Centers for Disease Control and Prevention (CDC): (i) severe illness requiring hospitalization in adults ≥21 years old; (ii) a positive SARS-CoV-2 test <12 weeks ago; (iii) severe organ dysfunction (excluding pulmonary manifestations); (iv) laboratory evidence of inflammation (e.g., elevated CRP, ferritin, D-dimer, or IL-6); and (v) absence of severe respiratory illness [3]. The patient rapidly improved with corticosteroid treatment; however, she presented again two months later with intermittent chest pain, with a workup identifying SCAD of the LAD with an apical aneurysm that was medically in accordance with the current best practices [10]. The systemic inflammatory response of MIS has repeatedly been described as having significant overlap with the proposed pathophysiology of Kawasaki disease [5], and the MIS-C variant has been reported to have a similar rate of coronary involvement [11], though only SCAD was noted in our patient during a coronary examination. Due to the paucity of MIS-A reports, the frequency and correlation of coronary involvement are not well-assessed, though cardiac dysfunction has been described in a majority of COVID-19 and MIS-A cases, including complications such as myocarditis, thrombosis, and arrhythmia, as well as persistent cardiac symptoms such as chest pain, shortness of breath, and palpitations [12,13]. Given the time between our patient’s MIS and SCAD presentations, it is difficult to establish clear causality. Finally, given that the incidence of SCAD is higher in young-to-middle-aged females with no conventional cardiovascular risk factors demographics shared by our patient-clinical suspicion for SCAD should be high following initial MIS management, though exact causation has yet to be reported. While further investigation is required to establish clear temporality, heightened clinical suspicion combined with improved patient education regarding symptomatic monitoring may improve diagnosis and treatment.

## Conclusions

Our case report highlights the importance of the potential delayed cardiac manifestations of COVID-19 infection. An unremarkable initial presentation followed by the development of MIS-A and subsequent SCAD illustrates the seriousness and unpredictability of COVID-19 infection, though further studies will be needed to establish causation. While efforts to understand these manifestations are needed to ensure a timely diagnosis and improve patient management, this case further highlights the importance of limiting the spread of COVID-19 as a means of limiting the development of serious complications in the first place, as even the most benign initial cases may produce serious outcomes.
